# Associations between perceived stress and health outcomes in adolescents

**DOI:** 10.1186/s13034-022-00510-w

**Published:** 2022-09-19

**Authors:** Frida Thorsén, Carl Antonson, Karolina Palmér, Rada Berg, Jan Sundquist, Kristina Sundquist

**Affiliations:** Center for Primary Health Care Research, Department of Clinical Sciences Malmö, Clinical Research Centre (CRC), Lund University, Skånes universitetssjukhus, Hus 28, våningsplan 11 Jan Waldenströms gata 35, 205 02 Malmö, Sweden

**Keywords:** Perceived stress, Adolescent, Cross-cultural, Sex differences

## Abstract

**Background:**

Adolescents are reporting increasing symptoms of anxiety, depression and somatization and an increase in perceived stress is a plausible explanation. The first aim of this study was to examine the occurrence of perceived stress and health outcomes in adolescents, and to evaluate if there are any sex differences. The second aim was to investigate if there is an association between perceived stress and the health outcomes and, if so, possible gender differences in this association. The third aim was to compare samples of adolescent girls and boys from two different European countries to enhance the generalizability of potential findings.

**Methods:**

The sample included 636 students from Sweden and Bulgaria, aged 15–16, 164 (58% males, 41% females, 1% not specified) from Sweden and 472 (71% males, 28% females, 1% not specified) from Bulgaria. Perceived stress and health outcomes were measured by the 14-item “Perceived Stress Scale” (PSS-14), and a shorter version of the questionnaire "Children and Young People in Skåne" (Folkhälsoenkäten, FHE), respectively. T-test and Chi^2^ and/or Fisher’s exact test was used to compare results between boys and girls from the PSS-14 and health outcomes. The association between PSS and the health outcomes was assessed using Spearman’s rank correlation and comparisons between boys and girls were calculated using linear regression.

**Results:**

There were significant associations between perceived stress and psychiatric symptoms in all groups. Adolescent girls in both Sweden and Bulgaria consistently reported higher levels of perceived stress and more psychiatric and somatic symptoms than the boys.

**Conclusions:**

Evaluating methods for lessening the perception of stress, and their clinical presentation, should be considered in order to reduce the occurrence of psychiatric symptoms in adolescents.

**Supplementary Information:**

The online version contains supplementary material available at 10.1186/s13034-022-00510-w.

## Background

An increase in psychiatric disorders has been reported worldwide since the 1990s [1], although this trend has been noticeable ever since the end of World War II [2–4]. This increase also includes younger individuals; depression is now the number one cause of illness and disability in adolescents in Europe, as well as globally [5]. In the European region, mental health and behavioural disorders account for approximately 30% of the total burden of disease among adolescents [5], whereof slightly more than half is related to internalizing mental health problems such as depression and anxiety [6].

The findings that adolescents are reporting increasing symptoms of anxiety, depression and somatization [6–11] is of concern. When these symptoms are intense, and appear often, the diagnostic criteria for psychiatric disorder are sometimes met [12]. In a survey from 2021 among 40 000 Swedish individuals 16–84 years of age, a total of 22% of young women and 11% of young men (16–29 years of age) reported such serious symptoms of reduced mental wellbeing that the symptoms were assessed to match the graveness of an established psychiatric disorder [13].

However, also when failing to fully meet the diagnostic criteria for disease, the complaints can still be severe enough to deserve attention [14]. Half of all people who develop psychiatric disorders have their first symptoms by the age of 14, and it is likely that adolescents who receive appropriate care may suffer less from psychiatric disorders throughout their life [5].

The largest increases in psychiatric and somatic symptoms have been observed among young women [8]. In addition, women are still more likely than men to exhibit psychiatric and somatic symptoms [8, 10]. Adolescent girls tend to report such symptoms more often than boys, while boys more often than girls report good self-rated health and high life satisfaction [8]. The same pattern, with girls reporting more psychiatric and somatic symptoms than boys, has been observed in several other studies [4, 9, 15], including studies using validated scales [10, 16].

As perceived stress and psychiatric and somatic symptoms are closely intertwined, influencing the magnitude of the multidirectional relationships, classic scientific methods used for determining causal relationships are difficult to interpret [17, 18]. However, it has been suggested that the reported increase in psychiatric and somatic symptoms might be due to an increase in perceived stress [6, 12], and correlations between perceived stress and reported health complaints, including psychiatric symptoms, have indeed been observed [10, 16].

Moreover, when asking adolescents themselves to reflect on the increase in such symptoms, they instinctively propose an increase in stress-exposure, composed of, e.g. high performance requirements in society, freedom of choice (with corresponding individual responsibility), media-exposure and schoolwork, as possible mechanisms [4].

Both psychiatric and somatic symptoms might therefore be interpreted as reactions to perceived stress [19]. Additionally, an individual perceives stress if the demands, in the individual’s experience, is greater than they believe they can manage. The definition does not take actual, objective conditions into consideration, nor the individual’s objective ability to handle the situation [20]. A certain exposure might thus not necessarily result in the same amount of perceived stress in different individuals or populations. Instead, in such a model, the development of symptoms would rather depend on the cumulative sum of the actual amount of stress-exposure, the individual’s perception of that stress, as well as the individual’s ability to handle the stress.

Although an increase in perceived stress over time is a plausible, and often suggested, explanation for the observed increase in psychiatric and somatic symptoms in society, research linking perceived stress and the occurrence of psychiatric and somatic symptoms is scarce. In the present study, the first aim was to examine the occurrence of perceived stress and several health outcomes in adolescents and to evaluate if there are any gender differences. The second aim was to investigate if there is an association between perceived stress and the health outcomes and, if so, possible gender differences in this association. The third aim was to compare samples of adolescent girls and boys from two different European countries to enhance the generalizability of potential findings.

## Methods

This study is part of a larger study aiming at comparing differences and changes in personality characteristics in two European countries over time, with data collected at two different time points with approximately 25 years in between. The current study is based on the most recent data collection that took place in 2015–2016. The participating schools were chosen from different areas of two cities to obtain a representative study sample from all social groups. The Swedish schools were situated in Västerås, a city with a population of around 145 000 individuals in 2015. The Bulgarian schools were situated in Sofia, which had a population of around 1.3 million inhabitants in 2015. Sweden and Bulgaria are different in several aspects but also similar, i.e. both countries are located in Europe, are relatively secular with a Christian past and with a language that is almost exclusively spoken within the country. The populations of Bulgaria and Sweden in 2016 were 7.2 and 9.9 million inhabitants, respectively [21].

All students, which were aged 15–16 years in the participating schools, received a written invitation in class, containing information about the study´s aims and procedures. Inclusion criteria were ability to read and write the national language; however, no student was excluded due to this. The students who wanted to participate signed a document of informed consent. The participation rate for the Swedish adolescents was 70% and for the Bulgarian adolescents it was 77%. The reasons why students did not answer the questionnaire could be sick-leave, non-approved absence from class, or that they actively chose not to participate in the survey.

The sample included 636 students, 15–16 years of age. Of these, 164 (58% boys, 41% girls) were from Sweden and 472 (71% boys, 28% girls) were from Bulgaria. We used a self-reported, dichotomous ("boy or girl") item for gender identity.

The data were collected between October and December 2016 in Sweden and between October 2015 and February 2016 in Bulgaria. The data were collected during a designated school lesson, and all the participating students answered the questionnaires individually, with a teacher present to clarify questions from the students if necessary. All questionnaires were completed anonymously.

## Questionnaires

### Perceived stress scale (PSS)

The measurements of perceived stress were based on the widely used 14-item Perceived Stress Scale (PSS-14), constructed as a five-point Likert-scale. Scores range from 0–56, with higher scores indicating greater perceived stress. The PSS focuses on how the individual experiences the situation and does not reveal anything about the objective magnitude of stressors. The underlying constructs of the PSS are interpreted and measured in the same way independently of gender [22–24]. Previous evaluations have demonstrated a high test–retest reliability and internal consistency [22, 25–27]. A review of 11 studies confirmed that the instrument has good internal consistency, reliability and good test–retest reliability over two days to four weeks [28]. Perceived stress measures are reported to have higher ecological validity than physiological response parameters and self-reported psychiatric symptoms, as it examines the extent to which a person subjectively experiences life situations as stressful, beyond the mere incidence of these specific events during a given period of time [24]. The PSS has been empirically validated in college students [28] and has been used in adolescents in clinical and normal populations to predict outcomes such as depression, anxiety and academic achievement [29]. The PSS-14 was translated to Swedish by Eskin & Parr in 1996 [23]. The translation was reported to have a high reliability in terms of internal consistency with an alpha coefficient of 0.82, i.e. well in line with the original coefficients of 0.84–86 acquired by Cohen in their original study [22]. The Bulgarian version of the PSS-14 has also been shown to have a high reliability with an alpha coefficient of 0.80 in a study from 2008 [26].

### Region Skåne´s Public Health Survey—Children and Young People in Skåne (In Swedish: Folkhälsoenkäten, FHE)

For measurement of different health outcomes, we used a shorter version of the same questionnaire that is used in Region Skåne´s Public Health Survey, Children and Young People in Skåne (Folkhälsoenkäten, FHE). The original FHE was constructed in 2012. It consists of six different sections: "Demographics", "Health and Well-being", "Life-style habits", "Social relations and safety", "School" and "Belief in the future" [30]. All items in the present study came from the 20-item section "Health and well-being". We selected all the items that were considered relevant for measurement of psychiatric and psychosomatic symptoms, in total eleven items.

It investigates living conditions, health outcomes and living habits among school students in grade six, grade nine and year two in high school. The survey contains questions about the student, family, health, accidents, leisure habits, eating habits, sleep, alcohol, tobacco, drugs, gambling habits, social relations, school and hope for the future. The questionnaire was tested and evaluated by school students in a pilot-study before broader use. The internal non-response rate is low, and the vast majority of items have an internal non-response rate below 5%. Several of the questions are also included in various national surveys, which allows for national comparisons [30]. The first comprehensive public health survey among children and young people was conducted in 2012 in collaboration between Region Skåne and the Municipality of Skåne. A follow-up was published in 2016 [31], and the latest report from the most recent survey conducted in 2021 is also available [32].

## Statistics

STATA version 14 (Stata Corp LP) was used for all statistical analyses.

The data obtained from the FHE is ordinal in its nature and, hence, no imputation could be made, nor could any measure of variability be reported. The scores from PSS are presented as means, with standard deviations (SDs) in brackets. We used mean imputation to account for missing data, using the individual´s mean, as this approach is intuitively easy to understand and compares well with both single regression and multiple imputation [33]. If less than (or equal to) 50% was missing of the 14 items in PSS, the missing items were imputed with the mean of the non-missing items for that individual. If more than 50% of the 14 items in PSS was missing, the whole scale was set to be missing. The Swedish adolescents had more missing values on PSS than the Bulgarian adolescents, and boys had more missing values than girls in both countries. The imputed cases had slightly lower PSS score than the complete cases. By stratifying for complete cases, imputed cases and missing cases (Table 1) we analyzed possible implications of the missing data. We repeated our analyses using only participants with complete PSS (Additional file 2: Table S2). The results were similar and did not change our conclusions.

**Table 1:** Descriptive data of participants stratified by complete cases, imputed cases and missing cases in PSS and FHE

	Sweden			Bulgaria		
	All	Boys	Girls	All	Boys	Girls
Included in the study, number (%)	164	95 (58)	67 (41)	472	333 (71)	132 (28)
Complete cases^a^ on PSS, number (%)	125 (76)	72 (76)	52 (78)	426 (90)	298 (89)	122 (92)
PSS total score, mean (SD)	27.3 (7.8)	26.1 (8.1)	28.9 (7.0)	24.0 (7.2)	22.7 (6.9)	27.0 (7.0)
Imputed cases^b^ on PSS, number (%)	23 (14)	12 (13)	11 (16)	43 (9)	32 (10)	10 (8)
PSS total score, mean (SD)	25.9 (8.2)	23.7 (9.8)	28.2 (5.5)	21.8 (7.1)	21.4 (6.9)	23.0 (8.2)
Missing cases^c^ on PSS, number (%)	16 (10)	11 (12)	4 (6)	3 (0.6)	3 (0.9)	0 (0)
Complete cases^d^ on FHE, number (%)	123 (75)	72 (76)	50 (75)	419 (89)	290 (87)	124 (94)
PSS total score, mean (SD)	27.1 (7.9)	25.7 (8.4)	28.9 (6.9)	23.6 (7.2)	22.3 (6.9)	26.7 (7.1)
Missing cases^e^ on FHE, number (%)	41 (25)	23 (24)	17 (25)	53 (11)	43 (13)	8 (6)
Number of missing questions, mean (min – max)	2.2 (1–11)	1.8 (1–8)	2.5 (1–11)	2.5 (1–12)	2.5 (1–12)	2.3 (1–7)
PSS total score, mean (SD)	27.1 (7.6)	25.9 (8.6)	28.3 (6.3)	25.1 (6.9)	24.9 (6.4)	26.7 (8.2)
Response sample, number (%)	147 (90)	84 (88)	63 (94)	462 (98)	330 (99)	132 (100)
PSS total score, mean (SD)	27.1 (7.8)	25.8 (8.4)	28.8 (6.7)	23.8 (7.2)	22.6 (6.9)	26.7 (7.1)

^a^All 14 items on PSS were complete

^b^If less than or equal to 50% missing of the 14 items in PSS, the missing items were imputed with the mean of the non-missing items

^c^If more than 50% missing of the 14 items in PSS, the scale was set to missing

^d^All 12 questions in the health questionnaire were complete

^e^At least one of the 12 questions was missing on FHE

^f^Complete and imputed cases in PSS and complete information on sex

T-test and Chi^2^ and/or Fisher’s exact test was used to compare results between boys and girls from the PSS total score and health outcomes. The strength and direction of association between PSS and the health outcomes was assessed using Spearman’s rank correlation and is presented as ρ = rho. Rho = 1 represents a maximal positive association, rho = 1 represents a maximal negative association, and rho = 0 represents no association at all. A sensitivity analysis was performed to account for clustered observations within classes using generalized estimating equations (GEE) that gave similar results.

The p-values for comparisons between boys and girls in the associations were calculated using linear regression and adding an interaction term: health outcome*sex. A similar model was used when examining possible differences between the two countries, using the interaction term health outcome*country (Additional file1: Table S1).

We used a basic significance level of p < 0.05 for all comparisons. We then used the Bonferroni method to correct for multiple comparisons. For the results in each table the significance level of α was divided by the number of tests performed. For example, in Table 2, with 26 different tests, a significance level of 0.05/26 ≈ 0.002 was used when interpreting the data. Using the Bonferroni method is a conservative strategy that leads to an increased risk for type II errors, and it has been argued that no correction for multiple analyses is needed when working with data obtained from actual observations [34]. However, we performed a Bonferroni correction where most of the results remained significant. Therefore, our conclusion remains unchanged even when using this more conservative method.

**Table 2:** PSS total score and health outcomes in Sweden and Bulgaria, separated and compared between boys and girls

	Sweden (n = 162)			Bulgaria (n = 465)		
	Boys (n = 95)	Girls (n = 67)	p-value^c^	Boys (n = 333)	Girls (n = 132)	p-value^c^
PSS total score, imputed^a^						
Number (%)	84 (57)	63 (43)		330 (71)	132 (29)	
Mean (SD)	25.8 (8.4)	28.8 (6.7)	0.02	22.6 (6.9)	26.7 (7.1)	< 0.0001
Range	2; 39	12; 42		2; 50	12; 44	
Quartiles^b^ (%)	8/39/52/13	2/38/57/10		8/68/22/2	2/57/39/2	
Health outcomes from questionnaire, number (%):						
How are you, in general?						
Very good	41 (44)	19 (30)	0.04	124 (38)	46 (35)	0.10
Good	40 (43)	27 (43)		143 (44)	52 (40)	
Reasonable	11 (12)	12 (19)		52 (16)	30 (23)	
Bad	1 (1)	4 (6)		4 (1)	2 (2)	
Very bad	1 (1)	1 (2)		2 (1)	0 (0)	
Do you feel content with yourself?						
Yes, mostly	55 (58)	32 (49)	0.09	160 (49)	52 (40)	0.14
Yes, sometimes	37 (39)	25 (38)		158 (49)	73 (56)	
No, almost never	3 (3)	8 (12)		7 (2)	5 (4)	
Do you feel stressed by your schoolwork?						
Not at all	14 (16)	4 (6)	0.01	93 (29)	24 (18)	0.002
A little	46 (51)	25 (39)		126 (39)	51 (39)	
Pretty much	25 (28)	22 (34)		64 (20)	22 (17)	
Much	5 (6)	13 (20)		42 (13)	35 (27)	

How often have you had the following problems in the last 6 months?						
Felt low						
Every day	2 (2)	8 (13)	< 0.0001	10 (3)	15 (12)	0.003
> 1 time/week	10 (11)	14 (22)		35 (11)	35 (27)	
About 1 time/week	7 (8)	11 (17)		46 (14)	37 (29)	
About 1 time/month	25 (28)	17 (27)		98 (31)	19 (15)	
Seldom/never	45 (51)	14 (22)		129 (41)	23 (18)	
Irritated/bad mood						
Every day	7 (8)	8 (13)	0.03	13 (4)	23 (17)	< 0.0001
> 1 time/week	19 (21)	12 (19)		86 (26)	47 (36)	
About 1 time/week	19 (21)	22 (35)		99 (30)	33 (25)	
About 1 time/month	23 (25)	9 (14)		64 (19)	18 (14)	
Seldom/never	23 (25)	12 (19)		67 (20)	11 (8)	
Anxious/worried						
Every day	3 (3)	3 (5)	0.01	11 (3)	14 (11)	< 0.0001
> 1 time/week	4 (4)	10 (16)		35 (11)	29 (22)	
About 1 time/week	10 (11)	11 (17)		61 (19)	44 (33)	
About 1 time/month	23 (26)	16 (25)		83 (25)	29 (22)	
Seldom/never	50 (56)	24 (38)		136 (42)	16 (12)	
Feeling dizzy						
Every day	1 (1)	2 (3)	0.05	13 (4)	7 (5)	0.001
> 1 time/week	4 (4)	5 (8)		27 (8)	16 (12)	
About 1 time/week	10 (11)	12 (19)		27 (8)	23 (18)	
About 1 time/month	20 (22)	17 (27)		67 (20)	29 (22)	
Seldom/never	55 (61)	27 (43)		195 (59)	56 (43)	

	Sweden (n = 162)			Bulgaria (n = 465)		
	Boys (n = 95)	Girls (n = 67)	p-value^c^	Boys (n = 333)	Girls (n = 132)	p-value^c^
Stomach ache						
Every day	2 (2)	3 (5)	0.03	5 (2)	4 (3)	< 0.000
> 1 time/week	2 (2)	7 (11)		16 (5)	16 (12)	
About 1 time/week	10 (11)	9 (14)		30 (9)	30 (23)	
About 1 time/month	30 (33)	31 (49)		86 (27)	56 (43)	
Seldom/never	48 (52)	13 (21)		180 (57)	23 (18)	
Headache						
Every day	2 (2)	2 (3)	< 0.0001	5 (2)	6 (5)	< 0.0001
> 1 time/week	4 (4)	9 (14)		34 (11)	33 (25)	
About 1 time/week	9 (10)	18 (29)		47 (15)	27 (21)	
About 1 time/month	32 (34)	21 (33)		97 (30)	33 (25)	
Seldom/never	46 (49)	13 (21)		136 (43)	31 (24)	
Restless sleep						
Every day	5 (5)	5 (8)	0.54	13 (4)	9 (7)	< 0.0001
> 1 time/week	5 (5)	4 (6)		18 (6)	14 (11)	
About 1 time/week	14 (15)	10 (16)		19 (6)	17 (13)	
About 1 time/month	17 (18)	12 (19)		49 (15)	27 (20)	
Seldom/never	51 (55)	31 (50)		228 (70)	65 (49)	
How often have you felt happy in the last 6 months?						
Every day	35 (38)	27 (43)	0.13	148 (45)	59 (46)	0.92
> 1 time/week	26 (28)	18 (29)		146 (45)	55 (43)	
About 1 time/week	17 (18)	13 (21)		21 (6)	10 (8)	
About 1 time/month	5 (5)	1 (2)		5 (2)	3 (2)	
Seldom/never	10 (11)	4 (6)		7 (2)	2 (2)	
How easy is it for you to talk to adults?						
Very easy	20 (22)	13 (20)	0.60	26 (8)	9 (7)	0.72
Easy	13 (15)	17 (27)		55 (17)	30 (23)	
Neither easy nor difficult	26 (29)	15 (23)		76 (24)	27 (21)	
Difficult	16 (18)	9 (14)		68 (21)	34 (26)	
Very difficult	14 (16)	10 (16)		96 (30)	30 (23)	

^a^If more than 50% missing, the scale was set to missing. If less than or equal to 50% missing, the missing items were imputed with the mean of the non-missing items

^b^PSS total score divided into quartiles, 0–13, 14–27, 28–41, 42–56

^c^P-value for test (t-test or chi^2^ test/fisher exact test) between boys and girls

## Results

A total of 165 students consented to participate in the study in Sweden. One boy was excluded because of an incomplete questionnaire. In total, 46 students had no information on class. Two individuals had missing information on sex. In total, nine classes from three different schools with, on average, 13 students in each class participated from Sweden. A total of 472 students consented to participate in Bulgaria. Seven individuals had no information on sex. In total, 26 classes from six different schools with on average 18 students in each class participated from Bulgaria (Table 1).

**Fig. 1:**
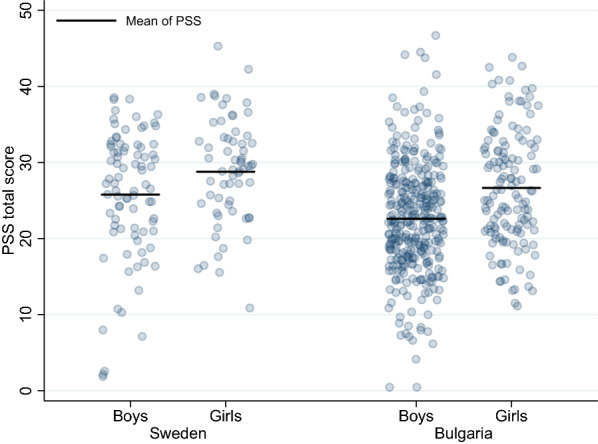
PSS total score for boys and girls in Sweden and Bulgaria (n_S_ = 147, n_B_ = 462)

The girls in both Sweden and Bulgaria reported higher scores on the PSS-scale than the boys within the same country (Table 1, Fig. 1 and Table 2).

A higher proportion of the Swedish adolescents, both boys and girls, reported PSS-values in the two upper quartiles (PSS total score divided into quartiles, 0–13, 14–27, 28–41, 42–56), compared with the Bulgarian adolescents (Swedish boys 65%, Bulgarian boys 24%) (Swedish girls 67%, Bulgarian girls 41%) (Table 2).

Girls in both Sweden (p = 0.01) and Bulgaria (p = 0.002) reported that they were more stressed by their schoolwork than the boys in the same country. Girls also reported feeling low (Sweden p < 0.0001, Bulgaria p = 0.003), being irritated (Sweden p = 0.03, Bulgaria p < 0.0001), being anxious (Sweden p = 0.01, Bulgaria p < 0.0001), feeling dizzy (Sweden p = 0.05, Bulgaria p = 0.001), having stomach ache (Sweden p = 0.03, Bulgaria p < 0.0001) and headache (Sweden p < 0.0001, Bulgaria p < 0.0001) to a higher extent than boys in both countries (Table 2). Most of these results remained significant also after the Bonferroni correction (i.e. a p-value below 0.002).

In both countries, girls reported feeling worse than the boys on the question “How are you, in general?”, which reached significance among the Swedish sample, (p = 0.04), and was a trend in the Bulgarian sample (p = 0.10), albeit without the Bonferroni correction (Table 2).

In Bulgaria, the girls reported higher frequency of restless sleep than the boys (p-value < 0.0001, Table 2). In Sweden, there was no difference between the sexes regarding restless sleep (p-value 0.54).

No significant difference between the sexes (Sweden p = 0.13, Bulgaria p = 0.92) was observed in either country on the questions “How often have you felt happy in the last 6 months?” or “How easy is it for you to talk to adults?” (Sweden p = 0.60, Bulgaria p = 0.72) (Table 2).

There was an association (assessed with Spearman’s rank correlation and expressed in ρ (rho)), between perceived stress and feeling stressed by schoolwork for both boys and girls, in both countries and in Bulgaria also with a Bonferroni correction calculated as 0.05/72 = 0.00069 for Table 3 (Swedish boys borderline significant, ρ = 0.22, p = 0.05, Bulgarian boys ρ = 0.30, p < 0.0001, Swedish girls ρ = 0.30, p = 0.02, Bulgarian girls ρ = 0.32, p = 0.0002). In all groups, the association between perceived stress and feeling low (Swedish boys ρ = 0.3, p = 0.008, Bulgarian boys ρ = 0.38, p < 0.0001, Swedish girls ρ = 0.43, p = 0.0005, Bulgarian girls ρ = 0.51 p < 0.0001), being irritated (Swedish boys ρ = 0.49, p < 0.0001, Bulgarian boys ρ = 0.37, p < 0.0001, Swedish girls ρ = 0.46, p = 0.0002, Bulgarian girls ρ = 0.54, p < 0.0001), significant also with a Bonferroni correction. The association with feeling dizzy (Swedish boys ρ = 0.26 p = 0.02, Bulgarian boys ρ = 0.35, p < 0.0001, Swedish girls ρ = 0.28, p = 0.03, Bulgarian girls ρ = 0.56 p < 0.0001) was significant in all groups, but after the Bonferroni correction only in the Bulgarian sample. There was also an association between perceived stress and being anxious (Swedish boys ρ = 0.22, p = 0.05, Bulgarian boys ρ = 0.37, p < 0.0001, Swedish girls ρ = 0.42, p = 0.0008, Bulgarian girls ρ = 0.54, p < 0.0001) and remained significant also with a Bonferroni correction in all groups except among the Swedish boys where it was only borderline significant. The associations were similar between boys and girls, which was confirmed by the non-significant interaction tests where all p-values were higher than 0.08. (Table 3).

**Table 3:** Association between PSS (total score) and health outcomes using spearman rank correlation, rho (ρ), in Sweden and Bulgaria, separated and compared between boys and girls

	Sweden (n = 147)			Bulgaria (n = 462)		
	Boys (n = 84)	Girls (n = 63)	Diff	Boys (n = 330)	Girls (n = 132)	Diff
	ρ (p-value)	ρ (p-value)	p-value^a^	ρ (p-value)	ρ (p-value)	p-value^a^
How are you, in general?						
Very good—very bad	0.13 (0.25)	0.38 (0.003)	0.10	0.44 (< 0.0001)	0.60 (< 0.0001)	0.08
Do you feel content with yourself?						
Yes, mostly—No, almost never	0.08 (0.45)	0.33 (0.01)	0.19	0.37 (< 0.0001)	0.49 (< 0.0001)	0.32
Do you feel stressed by your schoolwork?						
Not at all—Much	0.22 (0.05)	0.30 (0.02)	0.76	0.30 (< 0.0001)	0.32 (0.0002)	0.85
How often have you had the following problems in the last 6 months?						
Felt low						
Seldom or never—Every day	0.3 (0.008)	0.43 (0.0005)	0.72	0.38 (< 0.0001)	0.51 (< 0.0001)	0.49
Irritated/bad mood						
Seldom or never—Every day	0.49 (< 0.0001)	0.46 (0.0002)	0.35	0.37 (< 0.0001)	0.54 (< 0.0001)	0.15
Anxious/worried						
Seldom or never—Every day	0.22 (0.05)	0.42 (0.0008)	0.91	0.37 (< 0.0001)	0.54 (< 0.0001)	0.10
Feeling dizzy						
Seldom or never—Every day	0.26 (0.02)	0.28 (0.03)	0.51	0.35 (< 0.0001)	0.56 (< 0.0001)	0.46
Stomach ache						
Seldom or never—Every day	0.13 (0.25)	0.09 (0.48)	0.92	0.23 (< 0.0001)	0.34 (0.0001)	0.85
Headache						
Seldom or never—Every day	0.01 (0.90)	-0.21 (0.10)	0.26	0.22 (0.0001)	0.22 (0.01)	0.60
Restless sleep						
Seldom or never—Every day	0.05 (0.65)	0.28 (0.03)	0.58	0.18 (0.001)	0.33 (0.0001)	0.27
How often have you felt happy in the last 6 months?						
Every day—Seldom or never	0.009 (0.94)	0.48 (0.0001)	< 0.0001	0.29 (< 0.0001)	0.30 (0.0006)	0.54
How easy is it for you to talk to adults?						
Very easy—Very difficult	0.18 (0.11)	0.16 (0.23)	0.65	0.01 (0.79)	0.13 (0.14)	0.21

^a^P-value for test between boys and girls using linear regression with an interaction term (health outcome*sex)

There was no significant association between perceived stress and the question “-How easy is it for you to talk to adults?” for boys and girls in neither of the countries (Swedish boys p = 0.11, Bulgarian boys p = 0.79, Swedish girls p = 0.23, Bulgarian girls p = 0.14) (Table 3).

In Sweden, there was a significant difference (also after the Bonferroni correction) between girls and boys in the association between perceived stress and the question “-How often have you felt happy in the last 6 months?” (p-value from interaction test < 0.0001) where Swedish boys reported no such association (ρ = 0.009, p = 0.94) in contrast to the girls (ρ = 0.48, p = 0.0001).

No gender differences in the associations between perceived stress and any of the health outcomes were seen in Bulgaria (all p-values from interaction tests >  = 0.08) although nearly all associations were stronger for girls compared to boys (Table 3).

The Swedish boys reported their strongest significant association between perceived stress and being irritated/in a bad mood (ρ = 0.49, p < 0.0001), followed by (in descending order of association (but no longer significant after the Bonferroni correction) feeling low (ρ = 0.3, p = 0.008) and feeling dizzy (ρ = 0.26, p = 0.02) and with a borderline significant association between perceived stress and feeling stressed by schoolwork (ρ = 0.22, p = 0.05) and feeling anxious or worried (ρ = 0.22, p = 0.05). The Swedish boys reported no significant association between perceived stress and feeling content with oneself (p = 0.45), having stomach ache (p = 0.25), having headache (p = 0.90), experiencing restless sleep (p = 0.65), feeling happy (p = 0.94), general wellbeing (p = 0.25) or ability to talk to adults (p = 0.11) (Table 3).

The Swedish girls had the strongest significant association between perceived stress and feeling happy (ρ = 0.48, p = 0.0001), followed by (in descending order of association), being irritated/in a bad mood (ρ = 0.46, p = 0.0002), and feeling low (ρ = 0.43, p = 0.0005). The following associations were not significant after the Bonferroni correction; feeling anxious or worried (ρ = 0.42, p = 0.0008), general wellbeing (ρ = 0.38, p = 0.003), feeling content with oneself (ρ = 0.33, p = 0.01), feeling stressed by schoolwork (ρ = 0.30, p = 0.02), feeling dizzy (ρ = 0.28, p = 0.03), and experiencing restless sleep (ρ = 0.28, p = 0.03). They reported no significant association between perceived stress and having stomach ache (p = 0.48), having headache (p = 0.10) or ability to talk to adults (p = 0.23) (Table 3).

The Bulgarian boys had the strongest significant association between perceived stress and general wellbeing (ρ = 0.44, p < 0.0001), followed by (in descending order of association), feeling low (ρ = 0.38, p < 0.0001), being irritated/in a bad mood (ρ = 0.37, p < 0.0001), feeling content with oneself (ρ = 0.37, p < 0.0001), feeling anxious or worried (ρ = 0.37, p < 0.0001), feeling dizzy (ρ = 0.35, p < 0.0001), feeling stressed by schoolwork (ρ = 0.30, p < 0.0001), feeling happy (ρ = 0.29, p < 0.0001), having stomach ache (ρ = 0.23, p < 0.0001), and having headache (ρ = 0.22, p = 0.0001). The associations between PSS and experiencing restless sleep (ρ = 0.18, p = 0.001) was not significant after the Bonferroni correction. They reported no significant association between perceived stress and ability to talk to adults (p = 0.79). (Table 3).

The Bulgarian girls reported their strongest significant association between perceived stress and general wellbeing (ρ = 0.60, p < 0.0001), followed by (in descending order of association), feeling dizzy (ρ = 0.56, p < 0.0001), being irritated/in a bad mood (ρ = 0.54, p < 0.0001), feeling anxious or worried (ρ = 0.54, p < 0.0001), feeling low (ρ = 0.51, p < 0.0001), feeling content with oneself (ρ = 0.49, p < 0.0001), having stomach ache (ρ = 0.34, p = 0.0001), experiencing restless sleep (ρ = 0.33, p = 0.0001), feeling stressed by schoolwork (ρ = 0.32, p = 0.0002), feeling happy (ρ = 0.30, p = 0.0006). The association with having headache (ρ = 0.22, p = 0.01) was no longer significant after the Bonferroni correction. They reported no significant association between perceived stress and ability to talk to adults (p = 0.14) (Table 3).

When comparing the countries (Additional file1: Table S1), the Bulgarian adolescents had a tendency towards stronger associations (with higher rho-values and lower p-values) than Swedish adolescents for almost all health outcomes in both boys and girls, but the differences did not reach statistical significance. The only exceptions was that compared to the Swedish boys (ρ = 0.08, p = 0.45), the Bulgarian boys (ρ = 0.37, p < 0.0001) had a significantly stronger association between perceived stress and feeling happy (p < 0.0001), which remained significant also after the Bonferroni correction (calculated to 0.05/72 = 0.00069), whereas the association between perceived stress and general wellbeing (Swedish boys-Bulgarian boys p = 0.001, Swedish girls-Bulgarian girls p = 0.02), as well as between perceived stress and feeling content with oneself (Swedish boys-Bulgarian boys p = 0.003, Swedish girls-Bulgarian girls p = 0.02) was no longer significant after the Bonferroni correction.

## Discussion

The main findings of this study were the significant associations, in both boys and girls, in two different countries, between perceived stress and psychiatric symptoms. However, as the data is cross-sectional, we cannot establish the direction of the association, i.e. if an increase in perceived stress causes an increase in psychiatric symptoms or the opposite. Previous literature suggests that the relationship between stress and psychiatric symptoms is indeed bi- or even multi-directional [17, 18].

In accordance with earlier studies, adolescent girls in both Sweden and Bulgaria consistently reported higher levels of perceived stress and more psychiatric and somatic symptoms than the boys. The findings that girls reported higher demands and more psychiatric and somatic symptoms is well in accordance with earlier studies [4, 10, 35], including reports from larger population studies [12, 36–38].

Notably, all groups (both boys and girls and Sweden and Bulgaria) in the present study showed distinct associations between perceived stress and the psychiatric symptoms “*feeling irritated*”, “*feeling low*” and “*feeling anxious*”. All groups in this study also had an association between perceived stress and “*feeling stressed from schoolwork*”. It has previously been reported that girls feel stressed by their school work to a higher extent than boys, [31] and, in the present study, the girls in both countries followed this pattern. A higher proportion of high school girls experienced stress in their everyday lives compared with boys of the same age [30]. Before that age there does not seem to be a difference between girls and boys neither in stress-levels [30], nor in the reporting of psychiatric and somatic symptoms [38]. Differences in social constructions of gender [39, 40], a higher tendency towards performance-based self-esteem in girls [41] and girls taking on a larger social responsibility [42] have further been proposed to lie behind these gender differences. It has previously been suggested that the reported increase in psychiatric and somatic symptoms over time, in both boys and girls, might be due to an increase in perceived stress [6, 12]; additionally, the girls’ higher levels of perceived stress could possibly contribute to the gender difference in reported psychiatric symptoms observed in many earlier studies [12, 36–38].

The associations between perceived stress and the other health outcomes were more diverse. Regarding the somatic symptoms, all groups in this study had an association only between perceived stress and “*feeling dizzy*”. In Bulgaria, “*stomach ache*” and “*headache*” were also associated with perceived stress, but in Swedish adolescents, no such association was observed.

When examining the associations between perceived stress and health outcomes from a gender perspective, almost no significant gender differences in the associations between perceived stress and any of the health outcomes were observed. There was one exception, with Swedish girls reporting their strongest association between perceived stress and “*How often have you felt happy*”, whereas the Swedish boys showed no such association. Girls in Bulgaria generally tended to have stronger associations than the boys, although no statistically significant gender differences were observed.

The increase over time in self-reported psychiatric and somatic symptoms has previously been shown to be greater among adolescents in Sweden than among adolescents in many other European countries [31]. Almost 60% of the girls in Sweden have reported that they had at least two psychosomatic symptoms more than once a week during the past six months, compared with about 40 percent in many other European countries. Among the Swedish boys, a higher proportion also reported psychosomatic symptoms [43]. This corresponds with the findings in our study of a high proportion of the Swedish adolescents, both boys and girls, reporting higher levels of perceived stress than the Bulgarian adolescents. When dividing the total score from the Perceived Stress Scale (possible scores range from 0–56) into quartiles, 67% of the Swedish girls reported values in the upper two quartiles (> 28), compared to 41% of the Bulgarian girls. The corresponding numbers for the boys was 65% of the Swedish boys and 24% of the Bulgarian boys. For comparison, 24.4 was the mean score for both boys and girls when translating the scale to Swedish [23].

Interestingly, the Bulgarian adolescents showed a stronger (inverse) association between perceived stress and “*general wellbeing*” as well as between perceived stress and “*feeling content”* than the Swedish adolescents. The Bulgarian boys also showed a stronger (inverse) association between perceived stress and “*feeling happy*” than the Swedish boys. In essence, the Swedish adolescents reported higher levels of perceived stress but had weaker associations to those health outcomes measuring psychological wellbeing than the Bulgarian adolescents. It is possible that the Swedish adolescents, to a higher extent, confuse “being stressed” with “being successful” as it has been shown that in advanced economies being busy may correlate with elevated status, operating as a signal that an individual possesses desirable human capital capabilities and is in high demand [44]. However, a possible downside is increased frequency of being irritated, feeling anxious and feeling low.

The topic is undeniably quite complex, but we humbly suggest that the findings from this study may support the hypothesis that increased levels of perceived stress may partly explain the increasing frequency of reported psychiatric symptoms in society. Evaluating methods for lessening the perception of stress, both by reducing the actual stress burden (if possible), strengthening coping-abilities and factors of resilience, should be considered as methods for reducing the occurrence of psychiatric problems in adolescents.

## Limitations and strengths

The major limitation of this study is the cross-sectional study design. As with all cross-sectional studies, no conclusions on causality can be drawn. To complicate matters further, perceived stress and psychiatric and somatic symptoms are known to have a bi-or multidirectional relationship, often biased by the occurrence of additional resilience factors such as sleep quality.

The study was further based on self-reported data rather than objective measures. Perceived stress measures are, however, by nature self-assessed and reported to have higher ecological validity than, e.g. physiological response parameters, as they examine the extent to which a person subjectively experiences a situation as stressful, beyond the mere “objective” incidence of these specific events during a given period of time [24]. The PSS-scale is constructed with both positive and negative statements to minimize the risk for acquiescence bias, at the cost of increasing the risk of negative item bias. As the risk for negative item bias is known to decrease with increasing age, literacy and language skill [45] and since the adolescents had at least nine years of schooling and answered the test in their own language, this should not be a major issue. Unfortunately, we had no possibility to analyze the non-responders. It is quite possible that adolescents not present in school have a different profile of health than the attending ones. Comparing the population from the capital in one country to a medium-sized city in the other is not straightforward. However, we chose schools from different socioeconomic areas in both cities in order to enhance comparability.

There are also several strengths in the present study. The survey was made combining PSS, a reliable, well-validated scale that has previously been used in comparisons between countries, with questions frequently used in large, national studies. There is still a scarcity of studies on this age group using questionnaires that are well defined, validated, reliable, and that have previously been used in clinically relevant studies on adolescents, and this study therefore serves as an important contribution to previous studies. When selecting the study population we opted for schools from both countries that differed in terms of socioeconomic status and academic proficiency profile, with the aim of obtaining a sample as socioeconomically representative as possible. The participation rate of 70–77% could be regarded as acceptable if not good. By examining adolescents from two different European countries we enhanced the generalizability of the findings.

## Conclusion

We found significant associations between perceived stress and psychiatric symptoms in adolescents. Whereas experiencing psychiatric symptoms quite certainly increases the perception of stress, the results from this study may also support the idea that increased perception of stress might be a possible explanation for the observed increase in psychiatric symptoms in society. These findings should be useful for the adolescents themselves as well as for parents, schoolteachers, health care personnel and policymakers. Evaluating methods for lessening the perception of stress, and their clinical presentation, should be considered in order to reduce the occurrence of psychiatric symptoms in adolescents.

## Supplementary Information

**Additional file 1: Table S1.** Association between PSS (total score) and health outcomes using spearman rank correlation, rho (ρ), in boys and girls, separated and compared between Sweden and Bulgaria.

**Additional file 2: Table S2.** Repeated analyses of the results in Table 3 and Table S1 using only participants with complete PSS. Association between PSS (total score) and health outcomes separated and compared between boys and girls as well as between Sweden and Bulgaria.

## Data Availability

Data can be provided upon reasonable request but are not publicly available due to legal reasons.

## Abbreviations

FHE: Folkhälsoenkäten (a shorter version of the questionnaire "Children and Young People in Skåne")
GEE: Generalized Estimating Equations
PSS-14: Perceived Stress Scale (14-items)
SD: Standard Deviation
