# Supplementary material for: Associations between perceived stress and health outcomes in adolescents
Source: Child Adolesc Psychiatry Ment Health. 2022 Sep 19;16:75. doi: 10.1186/s13034-022-00510-w (PMC9487115; doi:10.1186/s13034-022-00510-w)
Supplement: Supplementary file 1 — Additional file 1: Table S1. Association between PSS (total score) and health outcomes using spearman rank correlation, rho (ρ), in boys and girls, separated and compared between Sweden and Bulgaria. [file 13034_2022_510_MOESM1_ESM.docx]

**Supplementary Table 1**: Association between PSS (total score) and health outcomes using spearman rank correlation, rho (ρ), in boys and girls, separated and compared between Sweden and Bulgaria

|  | **Boys** (n = 414) | | | **Girls** (n = 195) | | |
| --- | --- | --- | --- | --- | --- | --- |
|  | Sweden (n = 84) | Bulgaria (n = 330) | Diff | Sweden (n = 63) | Bulgaria (n = 132) | Diff |
|  | ρ (p-value) | ρ (p-value) | p-value^a^ | ρ (p-value) | ρ (p-value) | p-value^a^ |
| How are you, in general? |  |  |  |  |  |  |
| Very good – very bad | 0.13 (0.25) | 0.44 (< 0.0001) | 0.001 | 0.38 (0.003) | 0.60 (< 0.0001) | 0.02 |
| Do you feel content with yourself? |  |  |  |  |  |  |
| Yes, mostly – No, almost never | 0.08 (0.45) | 0.37 (< 0.0001) | 0.003 | 0.33 (0.01) | 0.49 (< 0.0001) | 0.06 |
| Do you feel stressed by your schoolwork? |  |  |  |  |  |  |
| Not at all - Much | 0.22 (0.05) | 0.30 (< 0.0001) | 0.58 | 0.30 (0.02) | 0.32 (0.0002) | 0.99 |
| **How often have you had the following problems in the last 6 months?** |  |  |  |  |  |  |
| Felt low |  |  |  |  |  |  |
| Seldom or never – Every day | 0.29 (0.008) | 0.38 (< 0.0001) | 0.26 | 0.43 (0.0005) | 0.51 (< 0.0001) | 0.25 |
| Irritated/bad mood |  |  |  |  |  |  |
| Seldom or never – Every day | 0.49 (< 0.0001) | 0.37 (< 0.0001) | 0.35 | 0.46 (0.0002) | 0.54 (< 0.0001) | 0.16 |
| Anxious/worried |  |  |  |  |  |  |
| Seldom or never – Every day | 0.22 (0.05) | 0.35 (< 0.0001) | 0.50 | 0.42 (0.0008) | 0.56 (< 0.0001) | 0.08 |
| Feeling dizzy |  |  |  |  |  |  |
| Seldom or never – Every day | 0.26 (0.02) | 0.23 (< 0.0001) | 0.24 | 0.28 (0.03) | 0.34 (0.0001) | 0.82 |
| Stomach ache |  |  |  |  |  |  |
| Seldom or never – Every day | 0.13 (0.25) | 0.22 (0.0001) | 0.24 | 0.09 (0.48) | 0.22 (0.01) | 0.40 |
| Headache |  |  |  |  |  |  |
| Seldom or never – Every day | 0.01 (0.90) | 0.18 (0.001) | 0.40 | -0.21 (0.10) | 0.29 (0.001) | 0.009 |
| Restless sleep |  |  |  |  |  |  |
| Seldom or never – Every day | 0.05 (0.65) | 0.18 (0.001) | 0.50 | 0.28 (0.03) | 0.33 (0.0001) | 0.49 |
| How often have you felt happy in the last 6 months? |  |  |  |  |  |  |
| Every day – Seldom or never | 0.009 (0.94) | 0.29 (< 0.0001) | < 0.0001 | 0.48 (0.0001) | 0.30 (0.0006) | 0.72 |
| How easy is it for you to talk to adults? |  |  |  |  |  |  |
| Very easy – Very difficult | 0.18 (0.11) | 0.01 (0.79) | 0.11 | 0.16 (0.23) | 0.13 (0.14) | 0.91 |

^a^P-value for test between countries using linear regression with an interaction term (health outcome*country)
