# Supplementary material for: Associations between perceived stress and health outcomes in adolescents
Source: Child Adolesc Psychiatry Ment Health. 2022 Sep 19;16:75. doi: 10.1186/s13034-022-00510-w (PMC9487115; doi:10.1186/s13034-022-00510-w)
Supplement: Supplementary file 2 — Additional file 2: Table S2. Repeated analyses of the results in Table 3 and Table S1 using only participants with complete PSS. Association between PSS (total score) and health outcomes separated and compared between boys and girls as well as between Sweden and Bulgaria. [file 13034_2022_510_MOESM2_ESM.docx]

**Table S2:** Repeated analyses of the results in Table 3 and Table S1 using only participants with complete PSS. Association between PSS (total score) and health outcomes separated and compared between boys and girls as well as between Sweden and Bulgaria.

|  | Table 3 | | | | | | | | | Table S1 | | | | | | | | |
| --- | --- | --- | --- | --- | --- | --- | --- | --- | --- | --- | --- | --- | --- | --- | --- | --- | --- | --- |
|  | **Sweden** | | | | **Bulgaria** | | | | **Boys** | | | | | **Girls** | | | |  |
|  | Boys | Girls | Diff | Boys | | Girls | Diff | Sweden | | | Bulgaria | Diff | Sweden | | Bulgaria | Diff |  |  |
|  | p-value^a^ | p-value | p-value^b^ | p-value | | p-value | p-value | p-value | | | p-value | p-value^c^ | p-value | | p-value | p-value |  |  |
|  |  |  |  |  | |  |  |  | | |  |  |  | |  |  |  |  |
| How are you, in general? | 0.17 | 0.003 | 0.65 | < 0.0001 | | < 0.0001 | 0.22 | 0.17 | | | < 0.0001 | 0.07 | 0.003 | | < 0.0001 | 0.02 |  |  |
|  |  |  |  |  | |  |  |  | | |  |  |  | |  |  |  |  |
| Do you feel content with yourself? | 0.36 | 0.009 | 0.38 | < 0.0001 | | 0.0001 | 0.59 | 0.36 | | | < 0.0001 | 0.009 | 0.009 | | < 0.0001 | 0.07 |  |  |
|  |  |  |  |  | |  |  |  | | |  |  |  | |  |  |  |  |
| Do you feel stressed by your schoolwork? | 0.13 | 0.007 | 0.63 | < 0.0001 | | 0.0006 | 0.97 | 0.13 | | | < 0.0001 | 0.82 | 0.007 | | 0.0006 | 0.39 |  |  |
|  |  |  |  |  | |  |  |  | | |  |  |  | |  |  |  |  |
| **How often have you had the following problems in the last 6 months?** |  |  |  |  | |  |  |  | | |  |  |  | |  |  |  |  |
|  |  |  |  |  | |  |  |  | | |  |  |  | |  |  |  |  |
| Felt low | 0.005 | 0.0006 | 0.80 | < 0.0001 | | < 0.0001 | 0.59 | 0.005 | | | < 0.0001 | 0.96 | 0.0006 | | < 0.0001 | 0.41 |  |  |
|  |  |  |  |  | |  |  |  | | |  |  |  | |  |  |  |  |
| Irritated/bad mood | < 0.0001 | 0.0007 | 0.24 | < 0.0001 | | < 0.0001 | 0.25 | < 0.0001 | | | < 0.0001 | 0.21 | 0.0007 | | < 0.0001 | 0.25 |  |  |
|  |  |  |  |  | |  |  |  | | |  |  |  | |  |  |  |  |
| Anxious/  worried | 0.06 | 0.002 | 0.77 | < 0.0001 | | < 0.0001 | 0.20 | 0.06 | | | < 0.0001 | 0.50 | 0.002 | | < 0.0001 | 0.26 |  |  |
|  |  |  |  |  | |  |  |  | | |  |  |  | |  |  |  |  |
| Feeling dizzy | 0.02 | 0.04 | 0.75 | < 0.0001 | | 0.0005 | 0.96 | 0.02 | | | < 0.0001 | 0.29 | 0.04 | | 0.0005 | 0.67 |  |  |
|  |  |  |  |  | |  |  |  | | |  |  |  | |  |  |  |  |
| Stomach ache | 0.24 | 0.51 | 0.61 | 0.0003 | | 0.003 | 0.70 | 0.24 | | | 0.0003 | 0.98 | 0.51 | | 0.003 | 0.32 |  |  |
|  |  |  |  |  | |  |  |  | | |  |  |  | |  |  |  |  |
| Headache | 0.53 | 0.11 | 0.64 | 0.0006 | | 0.001 | 0.74 | 0.53 | | | 0.0009 | 0.10 | 0.11 | | 0.001 | 0.01 |  |  |
|  |  |  |  |  | |  |  |  | | |  |  |  | |  |  |  |  |
| Restless sleep | 0.51 | 0.16 | 0.78 | 0.0009 | | 0.001 | 0.77 | 0.51 | | | 0.0009 | 0.39 | 0.16 | | 0.001 | 0.51 |  |  |
|  |  |  |  |  | |  |  |  | | |  |  |  | |  |  |  |  |
| How often have you felt happy in the last 6 months? | 0.51 | < 0.0001 | 0.0006 | < 0.0001 | | 0.0007 | 0.62 | 0.51 | | | < 0.0001 | 0.002 | 0.00003 | | 0.0007 | 0.28 |  |  |
|  |  |  |  |  | |  |  |  | | |  |  |  | |  |  |  |  |
| How easy is it for you to talk to adults? | 0.18 | 0.08 | 0.80 | 0.42 | | 0.12 | 0.31 | 0.18 | | | 0.42 | 0.40 | 0.08 | | 0.12 | 0.81 |  |  |

^a^P-value for association between PSS and health outcome within country and sex using spearman rank correlation

^b^P-value for test between boys and girls using linear regression with an interaction term (health outcome*sex)

^c^P-value for test between Sweden and Bulgaria using linear regression with an interaction term (health outcome*country)
